# Pulse oximeter accuracy and precision at five different sensor locations in infants and children with cyanotic heart disease

**DOI:** 10.4103/0019-5049.72642

**Published:** 2010

**Authors:** Jyotirmoy Das, Amit Aggarwal, Naresh Kumar Aggarwal

**Affiliations:** Department of Anesthesiology, Fortis Hospital, Shalimar Bagh, New Delhi, India; 1 Escorts Heart Institute and Research Center, New Delhi, India; 2 Department of Cardiac Anesthesiology, Fortis and Escorts Hospital, Vasant Kunj, New Delhi, India

**Keywords:** Accuracy, bias, precision, co-oximeter, pulse oximetry, SpO_2_, SaO_2_

## Abstract

Since the invention of pulse oximetry by Takuo Aoyagi in the early 1970s, its use has expanded beyond the perioperative care into neonatal, paediatric and adult intensive care units (ICUs). Pulse oximetry is one of the most important advances in respiratory monitoring as its readings (SpO_2_) are used clinically as an indirect estimation of arterial oxygen saturation (SaO_2_). Sensors were placed frequently on the sole, palm, ear lobe or toes in addition to finger. On performing an extensive Medline search using the terms “accuracy of pulse oximetry” and “precision of pulse oximetry”, limited data were found in congenital heart disease patients in the immediate post-corrective stage. Also, there are no reports and comparative data of the reliability and precision of pulse oximetry when readings from five different sensor locations (viz. finger, palm, toe, sole and ear) are analysed simultaneously. To fill these lacunae of knowledge, we undertook the present study in 50 infants and children with cyanotic heart disease in the immediate post-corrective stage.

## INTRODUCTION

To examine the accuracy and precision of pulse oximetry in cyanotic heart disease patients in the early post-operative period:

- When sensor is placed at five different locations (viz. finger, palm, toe, sole and ear).
- At low saturation states (SaO_2_*<*90).
- To identify the best sensor location.

## METHODS

After ethics committee approval, 50 children in the age group of 1 month to 7 years after various corrective surgeries for cyanotic congenital heart diseases in the early post-operative period were selected for this observational study.

### Exclusion criteria

- Core body temperature <35°C
- Children with diseases or conditions known to affect pulse oximeter accuracy, e.g. sickle cell disease, congenital methaemoglobinaemia etc.
- Lactate level >2 mmol/L
- High inotropic support (dopamine/dobutamine ≥5 *μ*/kg/min).

All the patients were on ventilatory support with an FiO_2_ of 0.6 and invasive arterial line in place. Measurements were taken within 3h of arrival in the intensive care unit (ICU) after corrective surgery.

The pulse oximeter used was a Philips M1020A pulse oximetry module (Philips Medical Systems, Eindhoven, Netherlands). An appropriate-sized pulse oximeter sensor (Phillips M1020A/ M1192A/ M1194A/ M1195A) was applied to the finger and palm of the same upper limb, on the toe and sole of the same lower limb along with the ear lobe. At the same time, an arterial blood sample was drawn. A single reading of SpO_2_ is taken in individual sensor locations after the pulse oximeters had achieved optimal plethysmographic signals and heart rates matching with the electrocardiogram monitor (CMS; Philips Medical Systems). The sensors were covered with black carbon paper, the overhead light was dimmed and ambient lights were reduced by screens to prevent interference. Simultaneously, an arterial blood gas (ABG) analysis was done for co-oximetric measurement of SaO_2_ using an ABL 800 FLEX ABG machine (Radiometer America Inc., Ohio, USA). Core temperature, arterial pressure, heart rate and ABG value of haemoglobin were recorded simultaneously.

The study population was further subdivided into Group A (SaO_2_<90%) and Group B (SaO_2_≥90%) to analyse changes in accuracy and precision at low oxygen saturation.

**Figure 1:**
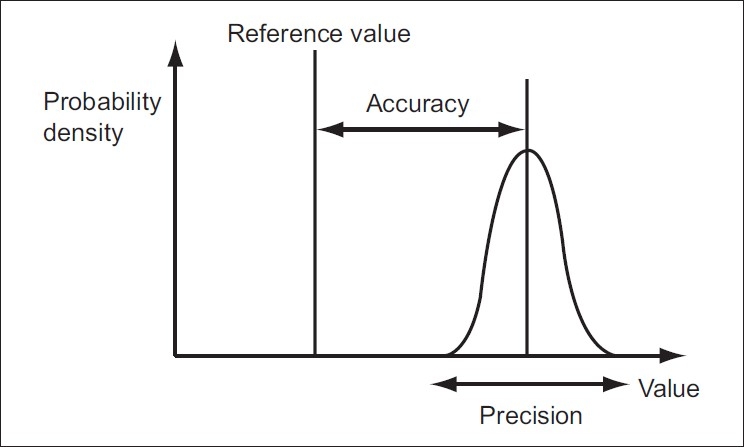
Accuracy is the proximity of measurement results to the true value. Precision is the repeatability or reproducibility of the measurement

Comparison of pulse oximetry reading (SpO_2_) with arterial oxygen saturation (SaO_2_) is reported in terms of bias and precision as described by Bland and Altman.[1] Bias is the difference between the specific body location SpO_2_ (finger, palm, toe, sole or ear) and SaO_2_ (i.e., SpO_2_-SaO_2_) and precision is the ±1 standard deviation of the difference.[1] A low bias in a sensor site implies that the pulse oximeter sensor gives a more accurate reading at that site and vice versa. Precision implies the reproducibility of the measurement [Figure 1]. Software SPSS 12.0 version was used for statistical analysis. One-way analysis of variance (ANOVA) test was used to compare the bias of two sensor sites statistically. The mean bias values of the two groups (Normoxaemic and Hypoxaemic) were compared using the unpaired *t*-test. *P*<0.05 was considered as significant.

## RESULTS

A total of 50 SaO_2_ measurements were obtained in 50 patients. The mean SaO_2_ was 96.1%±4.6%, with a range of 83–99.8%. Eight patients had SaO_2_ measurements <90% (Group A). The preoperative diagnosis of the study population is shown in Table 1.

Ear probes consistently showed the highest SpO_2_ results along with or without other sensor sites in 45 patients.

**Table 1:** Perioperative diagnosis of the study population with categorical distribution

Predominant preoperative finding	Number of patients
Double outlet right ventricle	8
Transposition of great arteries	18
Tetralogy of Falot	10
Miscellaneous	14

When the bias (i.e., SpO_2_- SaO_2_) is calculated in all the patients, it was observed that the bias is lowest with the sole sensor (-0.088) and highest in the ear sensor (1.572) (*P*=0.0049, using one-way ANOVA). Thus, in terms of accuracy (i.e., inverse of bias), we can say that the sole sensor is most accurate among the five sites of sensor location.

**Figure 2:**
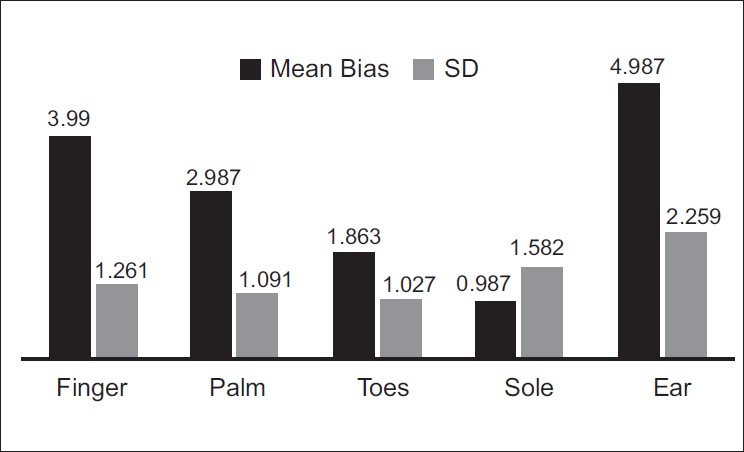
Pattern of mean bias and standard deviation in Group A

In Group A also, the sole sensor was found to have the least bias [Figure 2] and hence the most accuracy. Statistical correlation between the sole and the ear sensor was found to be highly significant (*P*<0.001) using the one-way ANOVA test.

**Figure 3:**
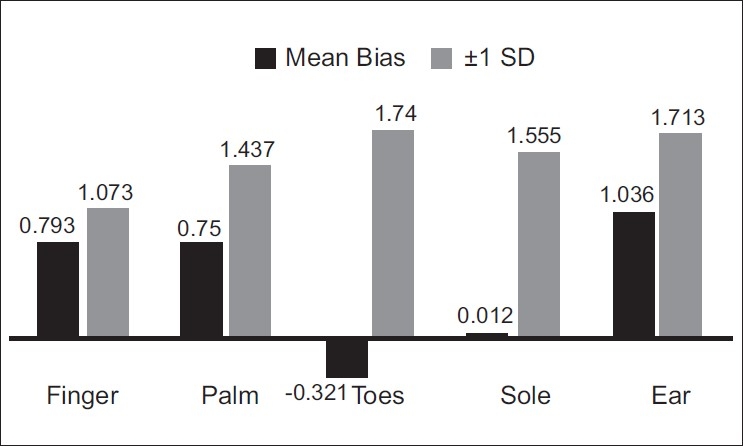
Pattern of mean bias and standard deviation in Group B

The same trend was maintained by the sole sensor even in Group B [Figure 3]. A statistically significant correlation was found between the sole and the ear sensors (*P*<0.0001) using one-way ANOVA.

It is noted that in Group A, the value of mean SpO_2_ (mean of all the five SpO_2_ readings) is always higher than the SaO_2_ [Table 2]. Thus, we can say that at low saturation states, SpO_2_ overestimates SaO_2_.

**Table 2:** Values of SpO_2_, SaO_2_ and mean bias in Group A

Mean SpO_2_	SaO_2_	Bias
89.4	85.8	3.6
91.6	89.6	2
85.6	83	2.6
90.8	89	1.8
93.8	89	4.8
86.6	84	2.6
91.4	89.5	1.9
87.6	85	2.6

Mean Bias 2.72, SD of Mean Bias 1.013

**Figure 4:**
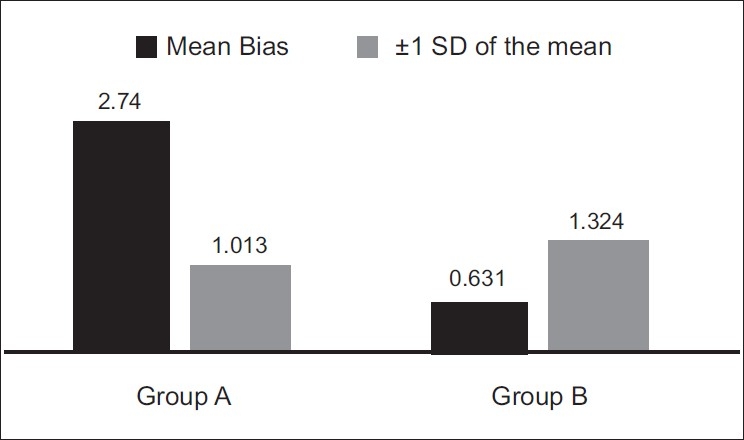
Comparison of mean group bias and its standard deviation between Group A and Group B

When the SaO_2_ value is deducted from this mean SpO_2_ value of a patient, we get the bias for that particular patient. When we consider the average of all the bias values in a particular group, we get the “mean bias” value for the group. The mean group bias in our study was found to be 0.631 and 2.74 in Group B and Group A, respectively [Figure 4] (*P*=0.0003 using unpaired *t*-test).

## DISCUSSION

Pulse oximetry in the words of Severinghaus and Astrup is “arguably the most significant technologic advance ever made in monitoring the well being of patients during anaesthesia, recovery and critical care”.[2] Pulse oximetry estimates arterial oxygen saturation by measuring the absorption of light of two wavelengths (approximately 660 nm and 940 nm) in human tissue beds. The amount of light absorption varies with the amount of blood in the tissue bed and the relative amounts of oxygenated and deoxygenated haemoglobin.[3] The accuracy of commercially available oximeters differ widely, probably due to the algorithm differences in signal processing.[4]

The aim of our study is to evaluate the accuracy and precision of pulse oximeter at five different sensor locations and to report any significant decline with hypoxaemia (SaO_2_<90%). Various studies reported a decline in accuracy and precision as the SaO_2_ decreases below various cut-off values (<90% or <80%)[5,6] The Philips M1020A module was selected for clinical use in our patients because of the accuracy and precision reported by Carter and colleagues.[7] They showed that the Philips M1020A was not affected by foetal haemoglobin (HbF). Different sensors also affect the accuracy of SpO_2_ measurements. Clayton and others found that the overall rankings were much better for the finger sensors in patients with poor peripheral perfusion.[8] Bell and others,[9] while comparing the traditional band-wrap disposable pulse oximeter sensor with the reusable clip-type sensor, found that the type of sensor selected has little effect on the accuracy of pulse oximetry in children.

Ambient light, skin pigmentation, dyshaemoglobinae-mia, low peripheral perfusion states and motion artefact can affect the performance of pulse oximeters.[10,11] The interference of ambient light can be overcome by simply wrapping the oximeter sensor in opaque material. Villanueva *et al*.[12] found that age, weight, core and skin temperature, haemoglobin concentration, pulse pressure and percent flow have little effect on the accuracy of pulse oximetry in children. At low levels of saturation (SaO_2_ below 80%), pulse oximetry is not as accurate as at higher saturations and overestimates the true value.[6] Although the exact mechanism is not known, various investigators have found that SpO_2_ overestimates SaO_2_ in polycythemia and underestimates SaO_2_ with anaemia.[13,14] This might explain the overestimation of SaO_2_ in the hypoxaemic group observed in the aforementioned studies as well as in our study. Sedaghat-Yazdi and others[15] while studying the effect of sensor location on pulse oximeter accuracy and precision in cyanotic children found that there are no significant differences in bias and precision between finger and toe sensors regardless of SaO_2_ values. They also found that sensor locations with the worst accuracy and precision were the sole and palm when SaO_2_ was <90%. This is contrary to the results of our study. Although the reason of our finding is not explainable clearly, factors like increased viscosity of blood, vascular and tissue changes due to clubbing in fingers and toes, etc. might play some yet unproven role. Better tissue perfusion in sole as compared to more peripheral sites like toe, finger and ear lobes might cause the sole sensor to perform better in terms of accuracy. In healthy volunteers, oximeters commonly have a mean difference (bias) of <2% and a standard deviation (precision) of <3% when SaO_2_ is 90% or above.[16,17] Comparable results have also been obtained in critically ill patients with good arterial perfusion.[18] However, the accuracy deteriorates when SaO_2_ falls to 80% or less (bias varies from –15.0 to 13.1 while the precision ranges from 1.0 to 16.0).[16]

## CONCLUSION

Cyanotic heart disease patients pose a unique dilemma in terms of the reliability and precision of the pulse oximetry readings and the determination of the best location of the sensor, especially in infants. An understanding of the bias and precision of the pulse oximetry at various sensor sites would go a long way in the effective management of patients with cyanotic heart disease in the perioperative period. We strongly recommend that clinicians should verify the measurements by a co-oximeter and evaluate the pulse oximetry sensor used with the particular body site reliability indices to avoid any unacceptable over- or underestimation of the SaO_2_. This becomes even more relevant in hypoxaemic patients with low SaO_2_ readings as the margin of safety is very small. We found that sole is the most accurate site of sensor location in cyanotic heart disease paediatric patients. We could also re-establish the finding that at low saturation states, pulse oximetry accuracy deteriorates and tends to overestimate the SaO_2_. In terms of reproducibility, the best sensor site could not be determined definitely and consistently in our study.
